# The Cholesterol-Lowering Effect of Alisol Acetates Based on HMG-CoA Reductase and Its Molecular Mechanism

**DOI:** 10.1155/2016/4753852

**Published:** 2016-10-30

**Authors:** Fei Xu, Hui Yu, Cai Lu, Jun Chen, Wei Gu

**Affiliations:** ^1^School of Pharmacy, Nanjing University of Chinese Medicine, Nanjing 210023, China; ^2^Jiangsu Hospital of Traditional Chinese Medicine (TCM), The First Affiliated Hospital of Nanjing University of Traditional Chinese Medicine (TCM), Nanjing 210029, China

## Abstract

This study measured the impact of alisol B 23-acetate and alisol A 24-acetate, the main active ingredients of the traditional Chinese medicine Alismatis rhizoma, on total cholesterol (TC), triglyceride (TG), high density lipoprotein-cholesterol (HDL-C), and low density lipoprotein-cholesterol (LDL-C) levels of hyperlipidemic mice. The binding of alisol B 23-acetate and alisol A 24-acetate to the key enzyme involved in the metabolism of TC, 3-hydroxy-3-methylglutary-coenzyme A (HMG-CoA) reductase, was studied using the reagent kit method and the western blotting technique combined with a molecular simulation technique. According to the results, alisol acetates significantly lower the TC, TG, and LDL-C concentrations of hyperlipidemic mice, while raising HDL-C concentrations. Alisol acetates lower HMG-CoA reductase activity in a dose-dependent fashion, both* in vivo* and* in vitro*. Neither of these alisol acetates significantly lower the protein expression of HMG-CoA. This suggests that alisol acetates lower the TC level via inhibiting the activity of HMG-CoA reductase by its prototype drug, which may exhibit an inhibition effect via directly and competitively binding to HMG-CoA. The side chain of the alisol acetate was the steering group via molecular simulation.

## 1. Introduction

Alismatis rhizoma is the rhizome of* Alisma orientale* (Sam.) Juzepcz and belongs to the Alismataceae family. Alismatis rhizoma is a diuretic agent of key importance, whose diuretic effect is related to the season of harvest, the medicinal parts, the processing method, the route of administration, and the species of the tested organism. Moreover, the diuretic effect of genuine Alismatis rhizoma is strongest when collected in winter, while spring collection results in slightly reduced effect. In addition to its salt solution, other processed products have an evident diuretic effect. Furthermore, an ethanol extract of Alismatis rhizoma and its triterpenes possess diuretic functions that reduce urinary protein. The triterpenes in Alismatis rhizoma are alisol A, Alisol B, alisol B 23-acetate, alisol A 24-acetate, and so on [1, 2]. Additionally, Alismatis rhizoma is a lipid-regulating Chinese traditional medicine that is commonly used as treatment for hyperlipidemia. Studies have shown that its main lipid-regulating active ingredients are alisol acetates, predominately alisol B 23-acetate, and alisol A 24-acetate. The clinical application of Alismatis rhizome has been limited by a lack of molecular studies of its molecular interaction mechanism [3–9]. The studies described in this paper investigated the different effects of alisol A 24-acetate and alisol B 23-acetate on total cholesterol (TC), triglyceride (TG), high density lipoprotein-cholesterol (HDL-C), and low density lipoprotein-cholesterol (LDL-C) of hyperlipidemic mice and obtained the lipid-regulating macroscopic rule of alisol acetates. In this study, we investigated the mechanism of alisol acetates on lowering TC level firstly, and the regulatory mechanisms of alisol acetates on TG, HDL-C, and LDL-C levels would be the future research content of our group. The main enzyme involved in the metabolism of TC is 3-hydroxy-3-methylglutaryl-coenzyme A (HMG-CoA) reductase. This enzyme facilitates cholesterol synthesis from the beginning by catalyzing the synthesis of mevalonate (MVA) from HMG-CoA, further generating TC via squalene. The decreased HMG-CoA reductase activity can effectively reduce TC generation [10–13]. Using the reagent kit method, HMG-CoA reductase activity was measured* in vivo* and* in vitro* using liver homogenates of hyperlipidemic mice before and after the addition of alisol acetates. The results indicate that alisol acetates might lower TC via inhibition of HMG-CoA reductase activity and that the prototype drug of alisol acetate inhibits HMG-CoA activity instead of its* in vivo* metabolites. Western blotting was used to measure the effects of alisol acetates on the HMG-CoA reductase protein expression of hyperlipidemic mice. The results showed that alisol acetates might not inhibit HMG-CoA reductase activity via downregulation of its protein expression. Instead, it might inhibit the HMG-CoA reductase effect by directly and competitively binding with it. The binding interaction of alisol acetates and HMG-CoA reductase was studied using a molecular simulation technique. This technique yielded the following parameters: the binding constant, binding energy, hydrogen bonding, hydrophobic/hydrophilic groups, electrostatic energy, and van der Waals forces. The interaction model of alisol acetate and HMG-CoA reductase was built. These experimental results were compared with the pharmacological results in order to determine the steering groups of this type of compound and the key amino acid residues of the enzyme. This could shed light on the cholesterol-lowering mechanism of alisol acetates at the molecular level. The results were invaluable for promoting the applications of Alismatis rhizoma in the clinical practice.

## 2. Materials and Methods

### 2.1. Reagents and Instruments

**Figure 1:**
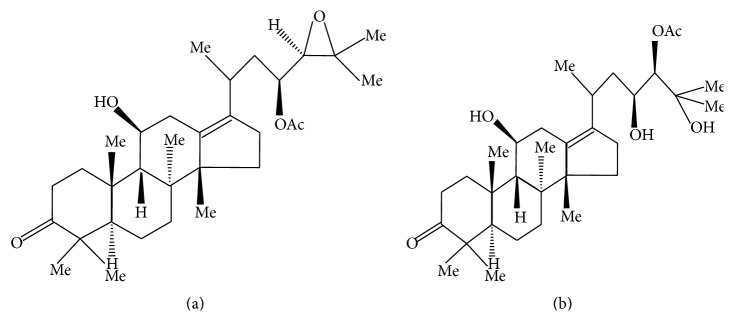
Molecular structures of alisol ((a) alisol B 23-acetate; (b) alisol A 24-acetate).

The main reagents were as follows: simvastatin (MSD Pharmaceutical Co., Ltd, Hangzhou, China), cholesterol (National Group Chemical Reagent Co., Ltd, Beijing, China), sodium deoxycholate (National Group Chemical Reagent Co., Ltd, Beijing, China), propylthiouracil tablets (Jinghua Pharmaceutical Co., Ltd, Nantong, China), lard (refined from the leaf lard purchased at the local market), polysorbate 80 (Tween 80, Qiangshun Chemical Reagent Co., Ltd, Shanghai, China), propylene glycol (Qiangshun Chemical Reagent Co., Ltd, Shanghai, China), basal diet (Slac Laboratory Animal Co., Ltd, Shanghai, China), radioimmunoprecipitation assay (RIPA) tissue cell lysis buffer (Solarbio, Beijing, China, cat number R0010), bicinchoninic acid (BCA) protein quantitative reagent kit (Thermo, MA, US, cat number PICPI23223), alisol A 24-acetate standard (Mansite Biological Technology Co., Ltd, Chengdu, China, batch number: MUST-15022104, 98% purity), and alisol B 23-acetate standard (Mansite Biological Technology Co., Ltd, Chengdu, China, batch number: MUST-149091210, with 98% purity). The structures of the alisol acetates are shown in Figure 1 [2, 14].

Further reagents were as follows: mice TG enzyme-linked immunosorbent assay (ELISA) reagent kits (Fengxiang Biotechnology Co., Ltd, Shanghai, China, batch number: 201207), mice HDL-C ELISA reagent kits (Fengxiang Biotechnology Co., Ltd, Shanghai, China, batch number: 201302), mice LDL-C ELISA reagent kits (Fengxiang Biotechnology Co., Ltd, Shanghai, China, batch number: 201301), mice TC ELISA reagent kits (Fengxiang Biotechnology Co., Ltd, Shanghai, China, batch number: 201202), HMG-CoA reductase activity assay kits (Genmed Scientifics Inc., Shanghai, China, batch number: 20080717), HMG-CoA reductase antibodies (Ray Biotech Inc., Atlanta, US, batch number: 168-10733), internal standard (glyceraldehyde-3-phosphate dehydrogenase [GAPDH]) protein antibody (CST, Boston, US, batch number: 5174), and nitrocellulose membrane (Millipore, Boston, US, batch number: HATF00010). All other chemicals used were of analytical grade and were obtained from China.

The following instruments were employed in this study: a high-speed ben centrifuge (Medical Analytical Instrument Factory, Shanghai, China, H1850), an electronic analytical balance (Sunny Hengping Scientific Instrument Co., Ltd, Shanghai, China, FA1104), pipettes (Jia'an Analytical Instrument Factory, Shanghai, China, WKYIII), a UV-Vis spectrophotometer (Persee General Instrument Co., Ltd, Beijing, China, TU-1901), a precision digital high-speed homogenizer (Shuoguang Electronic Technology Co., Ltd, Shanghai, China, SG-3048A), an electrophoresis apparatus (BIO-RAD, California, US, mini protean 3 cell), an electroporator (Jingmai Technology Co., Ltd, Dalian, China, PS-9), a microplate reader (Labsystems Multiskan MS, Finland, Model 352), a water bath (Leica, Germany, HI1210), and an imaging system (Tanon Co., Ltd., Shanghai, China, Tanon-5200).

### 2.2. Animals

Male clean-grade ICR mice (18–22 g) were purchased from the Animal Core Facility, Nanjing Medical University (license number: SCXK (Su)-2013-0005). The animals were placed in a clean-grade animal room at 20–24°C and 55%  ± 5% humidity. The light and dark cycles were twelve hours each. The mice were free-fed for one week prior to the experiments. The Animal Ethics Committee of Nanjing University of Chinese Medicine approved all protocols of animal experiments.

### 2.3. Measurements of TC, TG, HDL-C, and LDL-C Levels

#### 2.3.1. The Induction of an Animal Model of Hyperlipidemia [15–19]

A total of 99 male mice were randomly divided into nine groups according to their weight. Mice in one group, the normal blank group, were intragastrically fed distilled water (10 mL/kg) every morning. Mice in the other group were intragastrically fed lipid emulsion (10 mL/kg). The formulation of the lipid emulsion was as follows: 25% lard, 15% cholesterol, 10% polysorbate 80, 30% propylene glycol, 2% propylthiouracil, 2% sodium cholate, and 1% sugar. For five consecutive weeks, the mice were free-fed a basal diet alongside the intragastric administration of distilled water or lipid emulsion. The mice were fasted for twelve hours (water was permitted) after the last intragastric administration. Blood samples of mice were collected and their serums were separated. The TG, TC, HDL-C, and LDL-C concentrations in serums were measured according to the instructions of the corresponding reagent kits, making it possible to compare the blood lipid levels of the model and the blank groups. According to the results in Table 1, the TG, TC, and LDL-C of the model group increased while the HDL-C dropped. This suggests successful establishment of the hyperlipidemia models. The model animals were evenly divided into eight groups: model, simvastatin, alisol B 23-acetate (high, medium, and low dosage), and alisol A 24-acetate (high, medium, and low dosage).

#### 2.3.2. Drug Supply to Hyperlipidemic Mice [19]

The appropriate quantities of alisol A 24-acetate, alisol B 23-acetate, and simvastatin were weighed, dissolved in 0.3% sodium carboxymethylcellulose (CMC-Na) solution, and diluted to 26 mL.

The quantities administered intragastrically to each group were as follows: positive control group (simvastatin, 7 mg/kg/day), alisol A 24-acetate low-dose group (0.64 mg/kg/day), alisol A 24-acetate medium-dose group (1.28 mg/kg/day), alisol A 24-acetate high-dose group (2.56 mg/kg/day), alisol B 23-acetate low-dose group (0.64 mg/kg/day), alisol B 23-acetate medium-dose group (1.28 mg/kg/day), and alisol B 23-acetate high-dose group (2.56 mg/kg/day).

Each group was intragastrically fed 10 mL/kg of lipid emulsion every morning and treated with the corresponding drugs every afternoon for three weeks.

#### 2.3.3. Measurements of TC, TG, HDL-C, and LDL-C Levels

The mice were fasted for twelve hours (water was permitted) following the last dose of drug. Blood samples were collected and left to sit for 20–30 minutes. The serums were obtained by centrifuging the blood samples at 3000 r/min for 15 minutes. The collected serums were measured in accordance with the instruction of the TC, TG, HDL-C, and LDL-C reagent kits.

### 2.4. Measurement of* In Vivo/In Vitro* HMG-CoA Reductase Activity [19–25]

#### 2.4.1. Measurement of* In Vitro* HMG-CoA Reductase Activity

The establishment of the mouse model of hyperlipidemia was done according to Section 2.3.1. After the last intragastric administration (procedure described in Section 2.3.1), the mice were fasted for twelve hours (water was permitted) and then decapitated. Immediately after decapitation, the peritoneal cavities of the mice were rinsed with 10 mL of cold saline, while the livers of the mice were rapidly frozen in liquid nitrogen and stored at −70°C for further analysis.

At a temperature of 4°C, 0.5 g of each liver was weighed, placed in phosphate buffered saline (PBS, pH 7.2, 0.25 g/mL), and homogenized. Subsequently, the mixture was centrifuged for ten minutes at 4000 r/min and at a temperature of 4°C. The supernatant was then collected. On three separate occasions, 1 mL of the supernatant of the liver homogenate was collected and extracted with 10 mL of 95% ethanol at 60°C. This was followed by vacuum filtration. The residues were dissolved in PBS. The samples were adjusted to a certain concentration and stored at −70°C.

We added 0.04 mg/mL of simvastatin, 0.16 mg/mL, 0.08 mg/mL, and 0.04 mg/mL (high, medium, and low dosage) of alisol A 24-acetate, and alisol B 23-acetate to 5 mL of liver homogenate samples, respectively. After leaving the samples to stand for 24 hours, the HMG-CoA reductase activity was measured using the HMG-CoA reductase activity assay kit, in accordance with the instructions.

#### 2.4.2. Measurement of* In Vivo* HMG-CoA Reductase Activity

The induction of the animal model of hyperlipidemia was done according to Section 2.3.1. The intragastric administration was performed as described in Section 2.3.2. The mice were fasted for twelve hours (water was permitted) and decapitated after the last dose of the drug. The peritoneal cavities of the mice were immediately rinsed with 10 mL of cold saline, whereas the livers of the mice were rapidly frozen in liquid nitrogen and then stored for further analysis at a temperature of −70°C. At 4°C, 0.5 g of each liver were collected, placed in PBS (pH 7.2, 0.25 g/mL), and homogenized. The sample was subsequently centrifuged for ten minutes at 4000 r/min and at a temperature of 4°C. The supernatant was then collected. For each mouse, 1 mL of liver homogenate supernatant was extracted with 10 mL of 95% ethanol at 60°C on three occasions. This was followed by vacuum filtration. The residues were dissolved in PBS. The solution was adjusted to a certain concentration and stored at −70°C. The HMG-CoA reductase activity of the liver homogenate samples was measured in accordance with the instructions given in the HMG-CoA reductase activity assay kit.

### 2.5. Measurement of HMG-CoA Reductase Protein Expression Level via Western Blotting [26–29]

The hyperlipidemic induction of animal model of hyperlipidemia was built according to Section 2.3.1. The intragastric administration followed the procedure described in Section 2.3.2, whereas the liver homogenate samples were prepared as described in Section 2.4.2. The samples were stored at −70°C for further use.

In order to detect the protein concentration of the supernatant of each sample, the A_595 nm_ absorbance value was measured via the Bradford method and the calibration curve of the bovine serum albumin protein was plotted. An equivalent volume of 2 × sodium dodecyl sulfate (SDS) buffer was added to the liver homogenate sample. The mixture was denaturized at 100°C for three minutes. Subsequently, the protein was isolated using SDS-polyacrylamide gel electrophoresis (PAGE) and electrotransferred to the nitrocellulose membrane. The nitrocellulose membrane was removed and blocked between one and two hours in the tris-buffered saline with Tween 20 (TBST) solution with 5% skim-milk powder. Subsequently, the membrane was washed with TBST and placed into the primary antibody (1 : 2500 rabbit anti-rat HMG-CoA reductase polyclonal antibody) at 4°C overnight. The next day, the membrane was washed with TBST and blocked in the TBST solution with 5% skim-milk powder for two hours. The membrane was washed thrice with TBST for 10–20 minutes per wash. The membrane was incubated for one hour with alkaline phosphatase-conjugated mouse anti-rabbit IgG at a dilution of 1 : 500. Following incubation in the secondary antibody, the membrane was washed again and visualized using electrochemiluminescence (ECL). The membrane was placed in the antibody elution buffer for ten minutes and rinsed in TBST solution for five minutes. It was then blocked in the TBST solution with 5% skim-milk powder and shaken at room temperature for 30 minutes. The membrane was rinsed with TBST repeatedly and then incubated with primary antibody of internal standard GAPDH at a dilution of 1 : 1000. The membrane was subsequently washed and incubated with secondary antibody at a dilution of 1 : 250. Following washing, the membrane was visualized. The gray values of the filmstrips were measured using a gel imaging analysis system. The calculated gray level ratio (HMG-CoA reductase/GAPDH) represents the relative amount of HMG-CoA reductase protein expression.

### 2.6. Investigation of the Interaction between Alisol Acetates and HMG-CoA Reductase [30–35]

A molecular simulation was conducted. The initial molecular structures of the alisol acetates were generated using Discovery Studio 2.1 (DS2.1 Accelrys, US). The structure of HMG-CoA reductase was obtained from the crystal structure in the protein data bank (PDB) database (PDB code: 1HW9). HMG-CoA reductase is a glycoprotein with N-linked high-mannose oligosaccharides, consisting of four identical subunits with a molecular weight of 97 ku. Each subunit comprised 888 amino acid residues, with the phosphorylation site located at the 872nd amino acid. Each subunit featured three different domains: the N-terminal 339 (1–339) amino acids composed the transmembrane domain, the 110 (340–449) amino acids composed the junction domain, and the C-terminal 439 (450–888) amino acids composed the catalytic domain. The N-terminal crossed the endoplasmic reticulum membrane eight times and anchored to the membrane through short rings. The transmembrane domain was composed of 167 amino acid residues. As the four subunits were identical, according to its interaction with the drug, the A chain was selected as the receptor for docking simulation. Following the removal of water molecules, heteroatoms, and the multiconformations of the amino acid residues in the crystal structure, flexible docking in the Chemistry at HARvard Macromolecular Mechanics (CHARMM) force field was performed. Subsequently to the solvation calculation, the compensation ions Na^+^ and Cl^−^ were added to the system in order to simulate the environment in the human body. The most likely confirmation of the resulting complex was confirmed via minimization calculation.

#### 2.6.1. Molecular Simulation of Alisol B 23-Acetate and HMG-CoA Reductase

The alisol B 23-acetate was defined as a ligand. After docking, the LibDock score, the CDocker energy, the CDocker interaction energy, and the hydrogen bond formation of the ligand-receptor complex were comprehensively considered so as to determine the final steady conformation. This conformation was selected for further molecular dynamic simulation.

#### 2.6.2. Molecular Simulation of Alisol A 24-Acetate and HMG-CoA Reductase

Applying the method of Section 2.6.1 resulted in the final steady conformation of alisol A 24-acetate. This conformation was selected for further molecular dynamic simulation.

### 2.7. Data Analysis

The experimental data were analyzed using one-way analysis of variance (ANOVA) in SPSS version 15.0 (SPSS Inc., Chicago, USA). *P* values of less than 0.05 (*P* < 0.05) indicated statistically significant differences. The results were expressed in the form of mean ($\overset{-}{x}$) ± standard deviation (SD).

## 3. Results

### 3.1. Measurements of TC, TG, HDL-C, and LDL-C Levels

The blood lipid levels of the blank group, positive group, model group, alisol B 23-acetate high-, medium-, and low-dose groups, and alisol A 24-acetate high-, medium-, and low-dosage groups are shown in Table 1. This study revealed that the TC, TG, LDL-C, and HDL-C levels of the alisol A 24-acetate high-, medium-, and low-dose groups, as well as the alisol B 23-acetate high-, medium-, and low-dose groups, significantly differed from those of the model group. This suggests that alisol acetates could have a lipid-regulating effect and significantly decrease the TC, TG, and LDL-C concentrations of hyperlipidemic mice, while increasing the HDL-C concentrations.

### 3.2. Measurement of* In Vivo/In Vitro* HMG-CoA Reductase Activity

#### 3.2.1. Measurement of* In Vitro* HMG-CoA Reductase Activity

As Table 2 indicates, alisol acetates lower the HMG-CoA reductase activity dose dependently. The extent to which alisol B 23-acetate lowered HMG-CoA reductase activity exceeded the extent to which alisol A 24-acetate lowered the activity of this enzyme.

#### 3.2.2. Measurement of* In Vivo* HMG-CoA Reductase Activity

The results in Table 3 illustrate that alisol acetates lower the* in vivo* HMG-CoA reductase activity in a dose-dependent fashion, with a greater degree of enzyme inhibition for alisol B 23-acetate than for alisol A 24-acetate.

### 3.3. Measurement of HMG-CoA Reductase Protein Expression Level via Western Blotting

**Figure 2:**
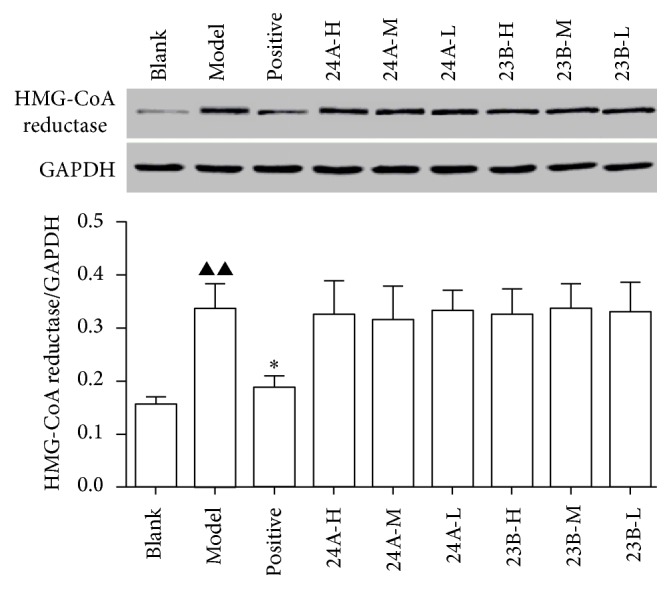
Results of the protein expression of HMG-CoA reductase in mice liver tissue protein (*n* = 11). Note: compared to the blank group, ^▲▲^ *P* < 0.01 and ^▲^ *P* < 0.05; compared to the model group, ^*∗∗*^ *P* < 0.01 and ^*∗*^ *P* < 0.05; 24A-H: alisol A 24-acetate high, 24A-M: alisol A 24-acetate medium, and 24A-L: alisol A 24-acetate low; 23B-H: alisol B 23-acetate high, 23B-M: alisol B 23-acetate medium, and 23B-L: alisol B 24-acetate low.

The western blotting results suggest that alisol acetates did not significantly decrease the protein expression of HMG-CoA reductase (Figure 2).

### 3.4. Investigation of the Interaction between the Alisol Acetates and HMG-CoA Reductase

#### 3.4.1. Molecular Simulation Results for Alisol B 23-Acetate and HMG-CoA Reductase

**Figure 3:**
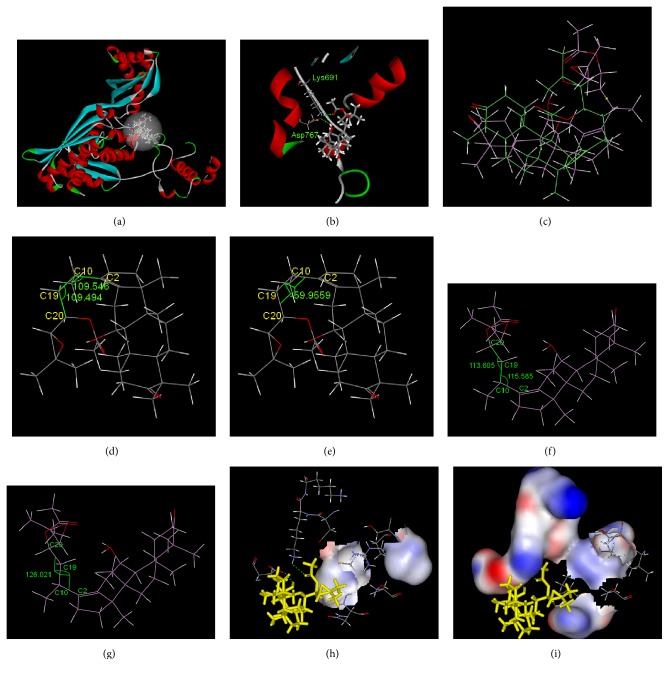
Interaction between alisol B 23-acetate and HMG-CoA reductase. (a) Overall pattern of interaction (alisol B 23-acetate is denoted by a bar chart). (b) Hydrogen bond graph of alisol B 23-acetate with Lys691 and Asp767 (the hydrogen bond is denoted by a dotted line). (c) Stacking chart of alisol B 23-acetate before and after docking (the initial structure is shown in green and the structure after docking is shown in purple). (d) Initial angle of alisol B 23-acetate C_2_–C_10_–C_19_ and C_10_–C_19_–C_20_. (e) Initial dihedral angle of alisol B 23-acetate C_2_–C_10_–C_19_–C_20_. (f) The angle of alisol B 23-acetate C_2_–C_10_–C_19_ and C_10_–C_19_–C_20_ after stacking. (g) The dihedral angle of alisol B 23-acetate C_2_–C_10_–C_19_–C_20_ after stacking. (h) Hydrophobic interface of the alisol B 23-acetate side chain and the peripheral amino acid residues after stacking (alisol B 23-acetate is denoted by yellow bars). (i) Hydrophilic interface of the alisol B 23-acetate side chain and the peripheral amino acid residues after stacking (alisol B 23-acetate is denoted by yellow bars).

The molecular simulation results for the interaction between alisol B 23-acetate and HMG-CoA reductase are depicted in Figure 3.  Figure 3(a) illustrates the overall pattern of the interaction. The interaction pocket where alisol B 23-acetate bound to HMG-CoA reductase contained the following amino acids: Leu521, Val522, Met523, Gly524, Ala525, Cys527, Glu528, Asn529, Val530, Met588, Thy589, Arg590, Gly591, Asp653, Ala654, Met655, Gly656, Met657, Asn658, Met659, Ile660, Ser661, Lys662, Gly663, Thy664, Glu665, Cys688, Asp690, Lys691, Lys692, Arg702, Lys704, Ile760, Try761, Ile762, Ala763, Cys764, Glu801, Ile802, Gly803, Thr804, Val805, Gly806, Gly807, Gly808, Thr809, Asn810, and Leu811. They were located in the catalytic domain constituted by the 439 (450–888) amino acids at the C-terminal. After entering the interaction pocket, alisol B 23-acetate interacted with the peripheral amino acid residues via hydrogen bonding and hydrophobic and hydrophilic effects. The hydrogen bond graph in Figure 3(b) shows two hydrogen bonds between alisol B 23-acetate and the amino acids Lys691 and Asp767.  Table 4 presents detailed information about the hydrogen bonding. The interaction energy between the hydrophobic amino acid residues and the small molecule was calculated to be −12.8 KJ/mol while the interaction energy between hydrophilic amino acid residues and the small molecule was calculated to be −135.3 KJ/mol. This indicates that the hydrophilic interaction between the small molecule and the peripheral amino acids was stronger.

After docking, the conformation of alisol B 23-acetate changed, as shown in Figure 3(c). The calculated root-mean-square deviation (RMSD) was 2.89 Ǻ. The conformation of the small molecule changed from the clustered sheet structure to the open structure. Before docking, the side chain and the ring structure of the small molecule folded, as shown in Figures 3(d) and 3(e). The angle between C_2_–C_10_–C_19_ was 109.5°, 109.5° between C_10_–C_19_–C_20_, and −59.9° between C_2_–C_10_–C_19_–C_20_. As shown in Figures 3(f) and 3(g), after docking, the angle between C_2_–C_10_–C_19_ was 113.6°, 115.6° between C_10_–C_19_–C_20_, and 126.0° between C_2_–C_10_–C_19_–C_20_. The angle between the parent ring and the side chain was significantly increased. The twisting of the dihedral angles resulted in the opening of side chains and insertion into the protein, which led to interactions. There was a hydrophilic ether bond on the side chain and the peripheral amino acids were mainly hydrophilic (Figures 3(h) and 3(i)). This created a hydrophilic environment around the side chains, culminating in stable binding. The energy of the van der Waals forces between them was calculated to be −151.9 KJ/mol, whereas the electrostatic energy was calculated to be −236.6 KJ/mol. As the electrostatic energy was lower than the energy of the van der Waals forces between the systems, the electrostatic interaction was the main interaction between this small molecule and the macromolecule. The binding energy (Δ*G*°) between the alisol B 23-acetate and the HMG-CoA reductase was calculated to be −103.3 KJ/mol.

#### 3.4.2. Molecular Simulation Results of Alisol A 24-Acetate and HMG-CoA Reductase

**Figure 4:**
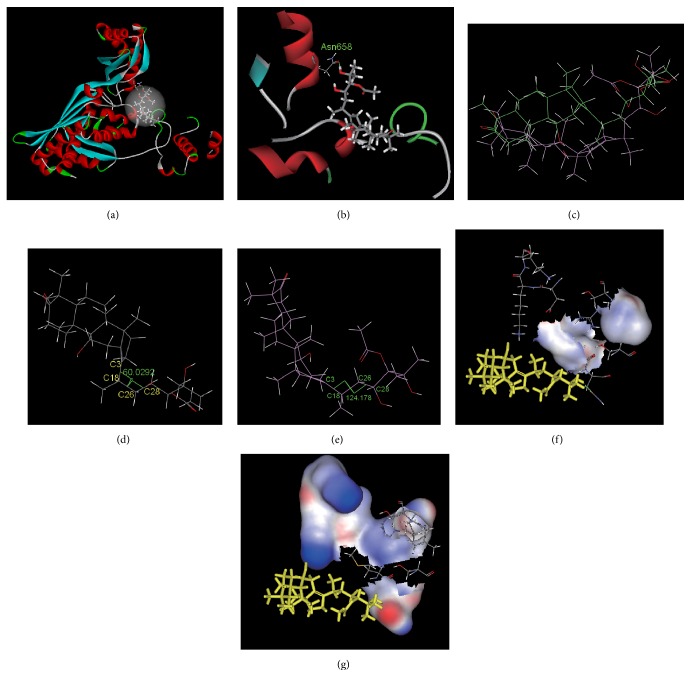
The interaction between alisol A 24-acetate and HMG-CoA reductase. (a) Overall pattern of interaction (alisol A 24-acetate is denoted by a bar chart). (b) Hydrogen bond graph of alisol A 24-acetate and Asn658 (the hydrogen bond is denoted by a dotted line). (c) Stacking chart of alisol A 24-acetate before and after docking (the initial structure is shown in green and the structure after docking is shown in purple). (d) The initial dihedral angle of alisol A 24-acetate: angle C_3_–C_18_–C_26_–C_28_. (e) The dihedral angle of alisol A 24-acetate: angle C_3_–C_18_–C_26_–C_28_ after stacking. (f) Hydrophobic interface of the alisol A 24-acetate side chain and the peripheral amino acid residues after stacking (alisol A 24-acetate is denoted by yellow bars). (g) Hydrophilic interface of the alisol A 24-acetate side chain and the peripheral amino acid residues after stacking (alisol A 24-acetate is denoted by yellow bars).

The molecular simulation results for the interaction between alisol A 24-acetate and HMG-CoA reductase are shown in Figure 4.  Figure 4(a) shows the overall interaction pattern of this compound and HMG-CoA reductase. The interaction pocket where it bound to HMG-CoA reductase contained the following amino acids: Tyr519, Ser520, Leu521, Val522, Met523, Gly524, Ala525, Cys527, Glu528, Asn529, Val530, Ile531, Gly532, Try533, Met588, Thy589, Arg590, Gly591, Asp653, Ala654, Met655, Gly656, Met657, Asn658, Met659, Ile660, Ser661, Lys662, Gly663, Thy664, Glu665, Cys688, The689, Asp690, Lys691, Lys692, Cys764, Gly765, Gln766, Asp767, Ala768, Ala769, Gln770, Asn771, Val772, Gly773, Ser774, Glu801, Ile802, Gly803, Thr804, Val805, Gly806, Gly807, Gly808, Thr809, and Asn810. There were 439 (450–888) amino acids in the catalytic domain at the C-terminal. After entering the interaction pocket, alisol A 24-acetate exhibited hydrogen bonding and hydrophobic and hydrophilic interactions with peripheral amino acid residues.  Figure 4(b) shows the hydrogen bond contact map. There was one hydrogen bond between alisol A 24 acetate and amino acid Asn658 (Table 4). The interaction energy of the hydrophobic amino acid residues and the small molecule was calculated to be 46.5 KJ/mol, whereas the interaction energy of the hydrophilic amino acid residues and the small molecule was calculated to be −102.3 KJ/mol. This suggests that the hydrophilic groups had a much stronger effect than the hydrophobic groups. The small molecule and the peripheral amino acids exhibited a stronger hydrophilic interaction, as indicated by the interface graphs (Figures 4(f) and 4(g)).

After docking, the alisol A 24-acetate conformation changed (Figure 4(c)). The RMSD was calculated to be 3.34 Ǻ. The conformation angle C_3_–C_18_–C_26_–C_28_ changed from 60.0° before docking to 124.2° after docking (Figures 4(d) and 4(e)). This suggests a large torsion of the small molecule side chain after docking, whereby the stretched open state changed to a ring-like sheet state. Consequently, it was difficult to insert the side chain into the protein and interact with the active pocket of the protein. This resulted in considerably lower binding relative to alisol B 23-acetate. The electrostatic energy of alisol A 24-acetate and HMG-CoA reductase was determined to be −88.6 KJ/mol while the energy of the van der Waals forces was determined to be −164.9 KJ/mol. The energy of the van der Waals forces was lower than the electrostatic energy, showing that the main interaction between this small molecule and the macromolecule was the van der Waals interaction. The Δ*G*° of alisol A 24-acetate and HMG-CoA reductase was calculated to be −2.3 KJ/mol.

## 4. Discussion

Various experimental and clinical studies focus on the blood lipid reducing effects of different solvent extracts (especially water and alcohol extracts) from Alisma orientale and its processed products [36–38]. However, the mechanisms underlying the effects of Alisma orientale constituents remain to be revealed. Alisma orientale may interfere with exogenous cholesterol absorption and endogenous cholesterol metabolism, thus reducing the level of TC. Moreover, alisol acetates may interfere with the metabolism of endogenous cholesterol [39].

Therefore, we studied the TC-lowering effect of alisol acetates based on HMG-CoA reductase and the underlying molecular mechanism. As the main source of endogenous cholesterol and one of the key enzymes in the TC metabolism, HMG-CoA reductase is the first described rate-limiting enzyme in cholesterol synthesis* in vivo*. Moreover, its activation directly affects the speed of cholesterol synthesis and the level of TC* in vivo*.

The TC-lowering effect and molecular mechanism of alisol acetates, the main active ingredient of Alismatis rhizoma, were investigated using the reagent kit method and western blotting technique combined with the molecular simulation technique. An interaction model was built for alisol acetate and the key enzyme involved in the metabolism of TC was HMG-CoA reductase. The present study reveals that alisol acetates can significantly lower the TC level of hyperlipidemic mice. The alisol B 23-acetate showed a higher extent of TC lowering than alisol A 24-acetate.

Consistent with the* in vitro* results, alisol acetates could also lower the* in vivo* HMG-CoA reductase activity in a dose-dependent fashion with a higher degree of enzyme inhibition for alisol B 23-acetate compared to alisol A 24-acetate. These results suggest that alisol acetates might lower TC levels via regulation of HMG-CoA reductase activity. The consistency between the results of the* in vivo* and* in vitro* experiments indicates that the alisol acetate prototype drug inhibits HMG-CoA reductase activity instead of the* in vivo* metabolites.

Revealed by the western blotting results, alisol acetates might inhibit the activity of HMG-CoA reductase by directly and competitively binding to HMG-CoA reductase rather than by downregulating its protein expression.

The molecular simulation technique was employed in order to investigate the interaction of alisol acetates and HMG-CoA reductase. The results (Figures 3 and 4 and Table 4) revealed the molecular mechanism of the interaction of alisol acetates and HMG-CoA reductase. Both alisol acetates bound to HMG-CoA reductase in their catalytic domain at the C-terminal. The alisol B 23-acetate formed two hydrogen bonds with HMG-CoA reductase. The hydrophilic interaction was stronger than the hydrophobic interaction, whereas the electrostatic interaction was stronger than the van der Waals forces. After the interaction, the structure of the small molecule dramatically changed from the original folded sheet to an open structure. Additionally, the side chain stretched and was inserted into the protein. The peripheral amino acids formed a hydrophilic environment and stably bound with it. In general, a hydrogen bond with a length of less than 3.5 Å and an angle of 90–180° is considered to be strong. These two hydrogen bonds were relatively strong and might be partly responsible for the torsion of the small molecule. The alisol A 24-acetate formed one hydrogen bond with the HMG-CoA reductase and this bond was weaker compared to those of alisol B 23-acetate. The hydrophilic interaction was stronger than the hydrophobic interaction and the van der Waals force was stronger than the electrostatic interaction. After the interaction, the molecule side chain had large torsion and changed from the original stretched open state to a ring-like sheet state, which was difficult to insert into the protein. The binding energy of alisol B 23-acetate and HMG-CoA reductase was lower than that of alisol A 24-acetate and HMG-CoA reductase, indicating that the binding of alisol B 23-acetate and HMG-CoA reductase was stable. According to the pharmacological results, alisol B 23-acetate lowered the HMG-CoA reductase activity more than alisol A 24-acetate. This suggests that the active center was the interaction pocket constituted by these amino acids and that the hydrogen bonds involving Lys691, Asp767, and Asn658 were the key amino acid residues for the interaction of this protein with alisol acetates.

The binding of alisol B 23-acetate and HMG-CoA reductase was considerably stronger than the binding of alisol A 24-acetate and HMG-CoA reductase. The relatively weak binding of alisol A 24-acetate and HMG-CoA reductase might be due to its distinct side-chain structure. The side chain of alisol B 23-acetate contained an ether bond, which was strongly hydrophilic and could be inserted into the hydrophilic environment of the protein to form strong interactions. Previous studies found that the side chains of alisol acetates were their active groups [40]. In the present study, this conclusion was validated. A folded side chain/parent ring bound more strongly to the macromolecule than an open side-chain/parent ring did. Thus, the side chain acts as the steering group in that it steers the interactions between alisol acetates and macromolecules. This might be the key group for interactions with macromolecules, as it had a decisive effect on the interactions of this type of compound with macromolecules. Our previous studies indicated that alisol B 23-acetate was partially converted to alisol A 24-acetate in the environment of the human stomach. However, the binding of alisol A 24-acetate and HMG-CoA reductase was weaker [14, 41, 42]. Therefore, it is advisable for drug developers to develop a dosage form that circumvents the conversion of alisol B 23-acetate in the stomach.

Previously published studies indicate that [43, 44] the mechanism of reducing the level of TC is that two acetyl CoA molecules condense to acetyl CoA under the catalysis of acetoacetyl-coenzyme A thiolase (AACT), acetyl CoA and acetyl CoA condense to HMG-CoA, and HMG-CoA reduces to MVA under the catalytic action of HMG-CoA reductase. MVA further generates TC with squalene. The high intracellular free fatty acid (FC) level stimulates microsomal acyl coenzyme A-cholesterol acyltransferase (ACAT) and promotes esterification of the cholesterol. Subsequently, cholesterol is stored in the form of cholesterol esters (CE), which is a feedback inhibition of HMG-CoA reductase synthesis, so that cholesterol synthesis rate-limiting enzymes will be inhibited, thus reducing intracellular cholesterol synthesis. This process features three important enzymes: AACT, HMG-CoA reductase, and ACAT. Whether AACT and ACAT are involved in the effect of alisol acetates will be the subject of intensive research in the future.

The results of the study indicate that alisol acetates can not only decrease TC level but also decrease TG and LDL-C levels and increase HDL-C level. Furthermore, a follow-up study is designed to investigate the regulatory mechanisms of alisol acetates on TG, HDL-C, and LDL-C.

## 5. Conclusion

The present study reveals that alisol acetates could significantly lower the TC level of hyperlipidemic mice. The effect of alisol B 23-acetate on HMG-CoA reductase was stronger than that of alisol A 24-acetate. Alisol acetates could lower HMG-CoA reductase activity in a dose-dependent fashion both* in vivo* and* in vitro*. Neither of these alisol acetates could significantly lower the protein expression of HMG-CoA. This suggests that alisol acetates lower the TC level via inhibiting the activity of HMG-CoA reductase via its prototype drug, which may have an inhibitory effect by directly and competitively binding with HMG-CoA. In the binding interaction of alisol acetate and HMG-CoA reductase via molecular simulation, the key amino acid residues of HMG-CoA reductase might be Lys691, Asp767, and Asn658. The side chain of the alisol acetate was the steering group. Furthermore, a folded side chain/parent ring bound more weakly to HMG-CoA reductase than an open side chain/parent ring. This study explored the TC-lowering mechanism of alisol acetates at the molecular level and presented guidance for its clinical use.

## Figures and Tables

**Table 1:** Blood lipid levels of each group of mice $(\overset{-}{x}\;\pm s$, *n* = 11).

Group	Animal count	TC (ng/mL)	TG (ng/mL)	HDL-C (ng/mL)	LDL-C (ng/mL)
Blank	11	8.94 ± 1.37	9.51 ± 1.52	68.93 ± 7.91	14.22 ± 4.57
Model	11	29.05 ± 1.49^▲▲^	30.59 ± 3.02^▲▲^	17.86 ± 5.59^▲▲^	51.71 ± 3.17^▲▲^
Positive	11	9.77 ± 1.44^*∗∗*^	10.98 ± 1.23^*∗∗*^	45.11 ± 5.72^*∗∗*^	21.11 ± 6.37^*∗∗*^
23B high	11	14.50 ± 1.91^*∗∗*^	14.08 ± 1.97^*∗∗*^	46.23 ± 8.48^*∗∗*^	26.39 ± 4.01^*∗∗*^
23B medium	11	23.10 ± 4.04^*∗∗*^	23.00 ± 1.67^*∗∗*^	37.56 ± 6.61^*∗∗*^	33.57 ± 3.72^*∗∗*^
23B low	11	24.30 ± 2.18^*∗∗*^	24.56 ± 4.18^*∗∗*^	29.08 ± 7.81^*∗∗*^	36.10 ± 4.63^*∗∗*^
24A high	11	18.70 ± 2.44^*∗∗*^	17.44 ± 2.24^*∗∗*^	42.89 ± 5.43^*∗∗*^	29.23 ± 6.33^*∗∗*^
24A medium	11	25.27 ± 5.31^*∗*^	26.79 ± 4.34^*∗*^	28.13 ± 5.53^*∗∗*^	36.92 ± 5.29^*∗∗*^
24A low	11	27.57 ± 1.68^*∗*^	27.15 ± 3.24^*∗*^	26.98 ± 3.77^*∗∗*^	38.57 ± 5.91^*∗∗*^

Note: 24A: alisol A 24-acetate; 23B: alisol B 23-acetate; compared to the blank group, ^▲▲^ *P* < 0.01 and ^▲^ *P* < 0.05; compared to the model group, ^*∗∗*^ *P* < 0.01 and ^*∗*^ *P* < 0.05.

**Table 2:** The activity of HMG-CoA reductase *in vitro *($\overset{-}{x}$  ±  *s*, *n* = 11).

Group	HMG-CoA reductase (U/L)
Blank	134.67 ± 39.91
Positive	164.29 ± 28.32^*∗∗*^
Model	497.88 ± 28.81^▲▲^
24A high	190.42 ± 30.92^*∗∗*^
24A medium	250.79 ± 16.35^*∗∗*^
24A low	335.15 ± 23.32^*∗∗*^
23B high	179.12 ± 41.59^*∗∗*^
23B medium	230.79 ± 16.64^*∗∗*^
23B low	327.59 ± 19.15^*∗∗*^

Note: 24A: alisol A 24-acetate and 23B: alisol B 23-acetate; compared to the blank group, ^▲▲^ *P* < 0.01 and ^▲^ *P* < 0.05; compared to the model group, ^*∗∗*^ *P* < 0.01 and ^*∗*^ *P* < 0.05.

**Table 3:** The activity of HMG-CoA reductase *in vivo* ($\overset{-}{x}$  ± *s*, *n* = 11).

Group	HMG-CoA reductase (U/L)
Blank	123.45 ± 25.34
Positive	200.21 ± 31.04^*∗∗*^
Model	490.12 ± 41.17^▲▲^
24A high	280.123 ± 29.15^*∗∗*^
24A medium	345.54 ± 32.34^*∗∗*^
24A low	401.31 ± 42.21^*∗∗*^
23B high	252.03 ± 29.31^*∗∗*^
23B medium	296.14 ± 32.18^*∗∗*^
23B low	390.82 ± 23.23^*∗∗*^

Note: 24A: alisol A 24-acetate and 23B: alisol B 23-acetate; compared to the blank group, ^▲▲^ *P* < 0.01 and ^▲^ *P* < 0.05; compared to the model group, ^*∗∗*^ *P* < 0.01 and ^*∗*^ *P* < 0.05.

**Table 4:** The length (Ǻ) and the angle of hydrogen bond formed between alisol acetates and HMG-CoA reductase.

Name	X–H⋯Y	*d* (X–H)	*d* (H⋯Y)	*d* (X⋯Y)	Angle XHY
Alisol B 23-acetate	Lys691: NZ-HZ1⋯O37	1.04	2.37	2.98	115.94
	Asp767: OD2-HH88⋯O2	0.96	2.40	3.24	144.90

Alisol A 24-acetate	Asn658: OD1-H5Z⋯O5	0.95	2.01	2.86	148.97
